# Label‐free Raman spectroscopic imaging to extract morphological and chemical information from a formalin‐fixed, paraffin‐embedded rat colon tissue section

**DOI:** 10.1111/iep.12194

**Published:** 2016-09-01

**Authors:** Riana Gaifulina, Andrew Thomas Maher, Catherine Kendall, James Nelson, Manuel Rodriguez‐Justo, Katherine Lau, Geraint Mark Thomas

**Affiliations:** ^1^ Department of Cell and Developmental BiologyUniversity College LondonLondonUK; ^2^ CoMPLEXUniversity College LondonLondonUK; ^3^ Biophotonics Research UnitGloucestershire Royal HospitalGloucesterUK; ^4^ Department of Statistical ScienceUniversity College LondonLondonUK; ^5^ Department of Gastrointestinal PathologyUniversity College HospitalLondonUK; ^6^ Spectroscopy Products DivisionRenishaw PlcWotton‐under‐EdgeUK

**Keywords:** biochemistry, formalin‐fixed paraffin‐embedded, haematoxylin and eosin, paraffin, principal component, Raman microspectroscopy, rat colon

## Abstract

Animal models and archived human biobank tissues are useful resources for research in disease development, diagnostics and therapeutics. For the preservation of microscopic anatomical features and to facilitate long‐term storage, a majority of tissue samples are denatured by the chemical treatments required for fixation, paraffin embedding and subsequent deparaffinization. These aggressive chemical processes are thought to modify the biochemical composition of the sample and potentially compromise reliable spectroscopic examination useful for the diagnosis or biomarking. As a result, spectroscopy is often conducted on fresh/frozen samples. In this study, we provide an extensive characterization of the biochemical signals remaining in processed samples (formalin fixation and paraffin embedding, FFPE) and especially those originating from the anatomical layers of a healthy rat colon. The application of chemometric analytical methods (unsupervised and supervised) was shown to eliminate the need for tissue staining and easily revealed microscopic features consistent with goblet cells and the dense populations of cells within the mucosa, principally via strong nucleic acid signals. We were also able to identify the collagenous submucosa‐ and serosa‐ as well as the muscle‐associated signals from the muscular regions and blood vessels. Applying linear regression analysis to the data, we were able to corroborate this initial assignment of cell and tissue types by confirming the biological origin of each layer by reference to a subset of authentic biomolecular standards. Our results demonstrate the potential of using label‐free Raman microspectroscopy to obtain superior imaging contrast in FFPE sections when compared directly to conventional haematoxylin and eosin (H&E) staining.

At approximately five to six feet in length, the colon is an extraordinary organ. Often perceived simply as a waste‐processing organ, it is in reality responsible for a diverse range of complex functions. It plays a crucial role in water and electrolyte absorption. Moreover, it is a vital habitat for a complex and dynamic community of approximately 100 trillion bacteria which are involved in a myriad of metabolic activities, trophic effects and immunological processes. As a result, common disorders of the colon such as inflammatory bowel disease (IBD) and cancer often lead to a wide range of complications and may have debilitating effects on the patients' quality of life or mortality (Nightingale 2001).

Current research into the various colonic pathologies and their treatment involves the routine use of animal models as substitutes for humans and provides an invaluable source of tissue material. Archived human material is also being extensively studied; however, the range of studies that can be carried out on such samples is limited. These tissue specimens contain an extraordinary breadth of information ranging from complex microanatomical features to elaborate cellular interactions, yet routine histological analysis captures only a very tiny portion of this. Collectively, much of the missing information could play crucial roles in our ability to make the most effective use of these tissues for diagnostic pathology, developmental biology (Donaldson *et al*. 2015), pathogenesis studies (Rijnierse *et al*. 2006) and drug efficacy studies (Fan *et al*. 2005; Taketani *et al*. 2014). Routinely, the bulk of such tissue specimens undergo preservation in formalin and subsequent paraffin embedding as this facilitates archiving and preservation of histomorphology but at the expense of the biochemical character (Butler *et al*. 2016). Very few specimens are subjected to freezing in liquid nitrogen which would better preserve both the anatomy and biochemistry.

The colon comprises of several distinct layers: the mucosa, submucosa, muscularis propria and serosa. The mucosa is the functional mainstay of the colon and is composed of a multitude of unique structures – the crypts of Lieberkühn. The colonic crypts are not static structures; rather, they are very dynamic environments maintained at equilibrium: large numbers of cells are continually replaced through stem cell division, differentiation and migration. These stem cells and proliferating cells are localized at the bottom half of the crypt and differentiated cells at the top; hence, in any given anatomical analysis the orientation of the tissue is critical if all the crypt cell populations are to be captured and visualized. The general colonic structure as well as the cellular composition of the functional regions has been well characterized (Humphries & Wright 2008). The structural organization of the colon is summarized in Table 1. Colonic dysfunction often involves only a small subset of cells; it is therefore increasingly important to first understand the normal biochemical states within the healthy tissues prior to detection of any telltale pathological changes.

**Table 1:** Correlating colonic anatomy to the structural organization and composition of a normal healthy colon

Colonic layer	Structural features	Composition
Mucosa	Crypts of Lieberkühn	Epithelial cells, goblet cells, enteroendocrine cells, stem cells
	Lamina propria	Connective tissue, plasma cells and lymphocytes
	Muscularis mucosa	Muscle cells
Submucosa	–	Connective tissue
		Blood vessels and lymphatics
		Lymphocytes
	Submucosal plexus	Nerve cells, connective tissue
Muscularis Propria	Inner circular muscle	Muscle cells
	Myenteric plexus	Nerve cells, connective tissue
	Outer Longitudinal muscle	Muscle cells
Serosa	–	Connective tissue
		Mesothelial cells

Some of the most frequently applied techniques in tissue analysis require the application of a range of stains, dyes or tags, haematoxylin and eosin (H&E) staining being the simplest and most common. H&E facilitates the staining of all cellular components and connective tissues irrespective of molecular composition and is the foundation technique in diagnostic histopathology. Various biological assays are also used to exploit the targeting properties of antibodies to tag specific proteins, and their localization can then be visualized by means of fluorescence or chromogenic enzymatic reactions (e.g. immunofluorescence (IF), immunohistochemical (IHC) analysis). Similarly, specific gene expression can also be identified by means of *in situ* hybridization (ISH), as with protein targeting a reporter tag must be conjugated to these probes; often, this is fluorescent. Over the years, such biomarker assays have grown to be an integral part of diagnostic pathology and clinical research; however, they possess certain key limitations. To maximize the sensitivity of diagnostic assays, a specific biomarker panel must first be carefully selected – poor or inappropriate selection fundamentally compromises analysis. Targeted, selective biomarker assays face further challenges arising from the underlying effects of chemical processing used for stabilization and preservation of the tissue, which can lead to selective antigenic loss and nucleic acid fragmentation (Srinivasan *et al*. 2002; Yaziji & Barry 2006).

Other more advanced tissue analysis techniques that do not rely on the quantification of previously identified biomarkers include matrix‐assisted laser desorption/ionization imaging mass spectrometry (MALDI IMS) (Shimma *et al*. 2007) and infrared (IR) spectral imaging (Nallala *et al*. 2014). However, MALDI IMS is limited to protein‐ and lipid‐based analysis and excludes the vital nucleic acid components. IR imaging on the other hand – although undoubtedly fast – is particularly susceptible to the problems of water contamination, limiting use to dried tissue samples. In contrast, Raman microspectroscopy, another advanced and sensitive technique, can be used to provide rapid, label‐free imaging of whole cells and tissues providing both spatial and chemical information simultaneously (Krafft *et al*. 2008; Bonnier & Byrne 2012). Unlike IR, water does not affect Raman spectroscopy making it an ideal technique for biological samples. The use of non‐ionizing and non‐mutagenic radiation wavelengths also makes Raman spectroscopy an attractive non‐destructive, non‐invasive techniques suitable for live cell and *in vivo* applications (Klein *et al*. 2012; Lui *et al*. 2012). By coupling a Raman system to a microscope set‐up, one can achieve submicrometre spatial resolutions (Maier & Treado 2004). Furthermore, many chemometric data analysis approaches have now been devised which allow cell/tissue classification to be carried out in a number of different ways (Krafft *et al*. 2008; Lloyd *et al*. 2012; Mavarani *et al*. 2013; Li *et al*. 2014).

There already exists a large body of work describing the application of Raman spectroscopy to colorectal tissue, most of which have focused on discrimination of tissues of colorectal cancer (CRC) and IBD (Molckovsky *et al*. 2003; Chowdary *et al*. 2007; Widjaja *et al*. 2008; Beljebbar *et al*. 2009; Piva *et al*. 2011). Most of these studies have focused on building robust CRC classifiers, but little attention has been paid to understanding the biochemical signals that originate from normal colon tissues and the underlying cell populations. The bulk of these studies are carried out on fresh/frozen tissues because these methods minimize sample handling and are thought to preserve the largest amount of biochemical information along with being free of any chemical contamination. Given the low sensitivity of earlier Raman instruments, this was thought to be essential because the tissue processing procedures used routinely in histopathology diminish Raman signal strengths (Devpura *et al*. 2013). Nevertheless, it is important to bear in mind that the bulk of all tissues removed for analysis undergo fixation and subsequent paraffin embedding. Seeing as this procedure ensures that adequate shelf life is maintained over a prolonged storage period, it is unreasonable to expect such routine pathology practice will undergo any significant changes in the coming years. In addition, we are yet to find a detailed Raman study summarizing the biochemical signals originating from the different cell populations lining colonic crypts as well as the surrounding tissue stroma from FFPE colonic samples. A study by Mavarani *et al*. (2013) evaluated the use of a 532 nm laser excitation source to identify more intricate structures within fixed colon sections, namely lymphocytes and erythrocytes. Again, this study was primarily focused around the characterization of cancerous colon tissue without full‐length normal crypt analysis.

The principle focus of this report was to demonstrate the power of Raman imaging in FFPE rat colon tissue sections. We aimed to provide an extensive biochemical characterization of the different anatomical layers present within the tissue. By utilizing a contiguously cut H&E stained section, we correlate the Raman generated images with the spatial distribution of the histological features. Moreover, we intended to correlate directly the biochemical signals obtained through Raman analysis with current knowledge of the chemical composition of the identified histological features. This, in turn, will provide a useful foundation for understanding the various disease processes explored through animal models – these include but are not limited to Crohn's disease, ulcerative colitis and cancer (Cassidy *et al*. 1980; Stĕpánková *et al*. 1996; Dieleman *et al*. 2004; Ganguly *et al*. 2015). To date, Raman has proven to be useful for the objective discrimination of Crohn's disease from ulcerative colitis, as well as *in vivo* efficacy testing of anti‐cancer drug treatments (Bi *et al*. 2011; Bielecki *et al*. 2012; Taketani *et al*. 2014).

## Materials and methods

### Sample preparation

Multiple colonic resections approximately 5 mm in length were excised from Wistar rats and fixed in standard 10% neutral buffered formalin for 24 h at room temperature. Tissues were then further processed in a Leica automated tissue processor TP1050 (Leica Biosystems GmbH, Nussloch, Germany) and embedded in standard laboratory histological paraffin wax. Sections were cut at 8 μm thickness and mounted onto calcium fluoride (CaF_2_) slides (Crystran Ltd., Poole, UK). Additional contiguous sections were cut at 3 μm, mounted onto a standard glass microscope slide and H&E stained for direct comparison. The 8 μm sections were subsequently deparaffinized in xylene and rehydrated in alcohol and water prior to being used for Raman imaging without any further treatment.

### Reference materials

A subset of reference biomolecules was selected and their distribution within the tissue investigated. Obvious features of the colon are muscle and nuclei from the dense cell population within the mucosa. To aid the identification of these components, reference spectra from muscle acetone powder and purified DNA were obtained. The submucosa, known to largely comprise of connective tissue, was referenced by authentic samples of collagen as well as hydroxyproline. Similarly, mucin contained within goblet cells was referenced by whole mucin, as well as the two most common sugars found in mucin – galactosamine and glucosamine. Reference spectra were also obtained from a number of lipids, namely phosphatidylcholine, phosphatidylserine, phosphatidylethanolamine, phosphatidic acid, cholesterol, oleic acid and palmitic acid as well as glycerol. Two carbohydrate molecules, glucose and fucose, were used as sugar‐based references. To reflect the dehydrated state of the tissue samples all references were measured dry unless they are naturally liquids, in which case they were used neat. Specific reference details can be found in Table 2.

**Table 2:** Summary of all the reference biomolecules as well as their origin

Reference compound	Supplier	Country
Paraffin wax (Tissue‐Tek^®^ II)	Sakura	UK
DNA (*herring testes*)	Sigma‐Aldrich	
Cholesterol		
Mucin type I (*bovine submaxillary glands*)		
Muscle acetone powder (*rabbit dehydrated muscle*)		
Glycerol		
Hydroxyproline		
Phosphatidylcholine		
Galactosamine		
Glucosamine		
Oleic acid		
Palmitic acid		
Phosphatidic acid		
Phosphatidylserine (*bovine brain*)		
Phosphoethanolamine (*egg yolks*)		
Collagen		
Fucose	Alfa Aesar	
Glucose	BDH Laboratory Supplies	

### Raman microscopic imaging

StreamLine^™^ Raman imaging was performed using the inVia Raman system (Renishaw plc, Wotton‐under‐edge, UK) coupled to a 785 nm laser excitation source and a Leica DM2500 microscope. A thermoelectrically cooled charge‐coupled device (CCD) camera was used for signal detection. StreamLine^™^ imaging uses a line geometry laser, which distributes the laser power along the length of a line rather than a single intense spot. A total laser intensity of approximately 50 mW was focused onto the sample through a 50× /NA 0.75 objective. A 1200 l/mm grating was used to disperse the light providing a spectral range of 400–1800 cm^−1^. A 2.8 μm step size was used with an integration time of 20 s producing a Raman map of the sample consisting of 81,405 unique spectra.

Several system checks were conducted before any data collection to ensure that the laser power and alignment were optimal. The system was then calibrated using a piece of silicon to the well‐characterized reference peak at 520.5 cm^−1^.

Biomolecular reference spectra were acquired on a Renishaw RA802 Raman microscope system, equipped with a 785 nm laser source emitting ~150 mW. This system was fitted with a 50× /NA 0.8 objective and provided a spectral resolution of ~2.6 cm^−1^ based on the full‐width half‐maximum of the silicon peak in the spectrum. Single‐point measurements were made with integration times ranging between 0.8 and 10 s, and this, however, was rectified through normalization.

### Data processing

#### Pre‐processing

The Raman maps were pre‐processed to reduce spectral variances originating from non‐chemical effects. This ensures that any instrumental artefacts are removed and the chemical information within the spectra is enhanced. Cosmic ray removal was conducted using the width of feature and nearest‐neighbour methods in WiRE 4 software (Renishaw plc).

Additional downstream processing was carried out in Matlab R2015b (MathWorks, Natick, MA, USA), including baseline correction by a third‐order polynomial and normalization, that is scaling, so that each spectrum had a mean intensity of zero and standard deviation equal to one. This ensures that each spectrum had a comparable influence on all the subsequent statistical analysis. For spectral analysis carried out in WiRE 4, baseline correction and normalization was conducted in Matlab R2015b, and the corrected spectra were then reimported into WiRE 4 for spectral analysis.

#### Principal component analysis

Principal component analysis (PCA) was used as an unsupervised multivariate data analysis technique that highlights the important differences in the tissue without prior knowledge of tissue biochemistry. PCA is a data reduction technique that condenses the spectral data to a set of variables, known as principal components (PCs). The first component explains the greatest variance in the data set, with subsequent components representing the highest remaining variance where they are orthogonal to the preceding components. Inspection of the individual loadings of the PCs enables us to gain an understanding into the specific biochemical information responsible for the variance observed. Coloured images are then generated from the PCs to reveal the spatial organization of the elements isolated as principal components. In our images, pixels with a high positive score for a given PC are assigned a false colour while pixels with high negative score are assigned a second colour, where the colour intensity corresponds to the relative contribution of the score.

PCA was carried out using both WiRE 4 and Matlab R2015b. Matlab facilitates the implementation of linear regression analysis following PCA as well as directly matching the spectral similarity of reference biomolecules. WiRE allows the generation of superior composite score images that can be compared to H&Es with relative ease. Using both platforms, we were able to perform comprehensive analysis of the image data. Prior to the analysis, using either platform spectra were mean‐centred and spectrum‐centred within the given software.

In this work, we have chosen to analyse four PCs that were considered to be the most histologically significant. These four PCs and a further six (the first ten PCs) account for 82% of the total spectral variance present in our data and can be found in Supporting Information.

#### Linear fit of the PCA

To explore the molecular basis of the difference underpinning the PCA, we fit a linear combination of reference spectra to each component. That is, we formulated each PC as:

$$p=w^{\left(1\right)}s^{\left(1\right)}+w^{\left(2\right)}s^{\left(2\right)}+\cdots +w^{\left(n\right)}s^{\left(n\right)},$$

where *w* ^(*i*)^ is the weight of reference spectrum *s* ^(*i*)^ in the linear sum.

In this way, we can establish which biomolecules contribute most strongly to each PC. Note that *w* ^(*i*)^ can be positive or negative – the specific sign does not denote the importance of the biomolecule; rather, it is |*w* ^(*i*)^|, the absolute magnitude of *w* ^(*i*)^ that does so. Furthermore, the negative or positive assignments of the linear fit bars are in line with the directionality of the peak assignments across the PC loadings.

The value of *w* ^(*i*)^ for each PC was determined by implementing least squares linear regression.

#### Spectral similarity

To determine the similarity between each pixel of the tissue map and each reference spectrum, we computed the cosine similarity, defined as:

$$\theta =\frac{m\cdot s}{\left|m||s\right|}=\frac{\sum_{i}m_{i}s_{i}}{\sqrt{\left(\sum_{i}m_{i}^{2}\right)\left(\sum_{i}s_{i}^{2}\right)}},$$

where *s*_*i*_ denotes the Raman intensity at Raman shift *i* in the reference spectrum and *m*_*i*_ denotes the Raman intensity at Raman shift *i* in the tissue map spectrum.

The similarity score is bounded such that −1 ≤ θ ≤ 1. A value of θ = 1 implies that the two spectra are identical, that is, *m *= *s*; if θ = −1, then the two spectra are opposite, that is, *m *= −*s*. A value of θ near zero indicates no correspondence between *m* and *s*.

As such, a large positive value of θ suggests that the pixel constitutes, to some degree, the reference spectrum. By plotting the value of θ across the whole map, we can determine which areas of the tissue are most similar to the reference spectrum at hand, and thereby deduce the distribution of that reference biomolecule within the tissue. The accuracy of this technique can be easily validated by visually comparing an average of the 100 best matching tissue spectra to the reference spectrum.

A background mask was applied to prevent the reference spectra from being matched against pixels associated with the backing substrate (CaF_2_ slide). To choose which pixels to discard, we first formed an average ‘background’ spectrum from a separate Raman map that contained only a CaF_2_ slide. We then computed the spectral similarity between all pixels of our Raman map and the average background spectrum. Any pixels with a similarity value greater than some threshold τ were discarded, ensuring only biological tissue was analysed. For this work, the threshold was determined to be τ = 0.98.

### Ethical approval statement

Research was conducted in full compliance with the UK Animals (Scientific Procedures) Act 1986 (A(SP)A).

## Results

### Rat colon H&E overview

**Figure 1:**
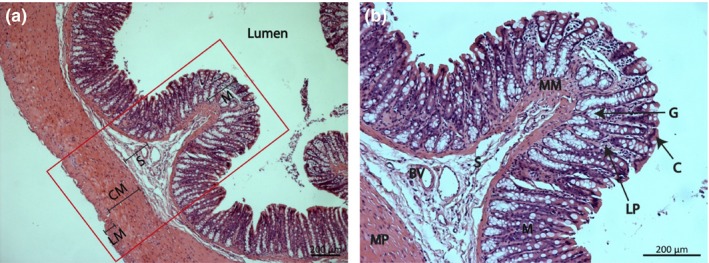
Transversely cut H&E‐stained rat colon section. (a) LM, outer longitudinal muscle; CM, inner circular muscle; S, submucosa; M, mucosa. Red box indicates the area that underwent Raman analysis. (b) Close‐up of a mucosal fold: M, mucosa; MM, muscularis mucosa; S, submucosa; BV, blood vessel; MP, muscularis propria; LP, lamina propria; G, goblet cells; C, colonocytes.

All the main layers of the colon are clearly defined with the thickest regions composed of the mucosa and muscularis propria. The outermost muscularis propria exhibits two distinctly orientated muscular layers: (i) inner circular muscle and (ii) outer longitudinal muscle (Figure 1a). The submucosa exhibits a very sparse distribution of connective tissue fibres with several embedded lymphocytes and blood vessels. The cells of the mucosa are very densely packed with very little visible lamina propria. The epithelial cells that line the crypts of Lieberkühn are best resolved at the luminal edge of the mucosa, whereas the larger goblet cells are the most easily identified feature (Figure 1b).

### Principal component analysis

PCA is an unsupervised multivariate analysis technique that allows an effective reduction of a large and complex spectral data set to a few most meaningful components. These components capture most of the variance within the data set, and they can then be tentatively assigned to a specific biological component present in abundance within a given anatomical region. Note that these components do not represent pure biochemical components but rather a mixture of the most abundant and discriminatory components. This technique provides a method of identification by reducing the subjectivity and speculation inherent in any process of manually selecting from amongst chemical reference spectra. Up to ten components were hypothesized to contain the most relevant biochemical information relating to the biochemical variance of the layers within the sample. However, only four components considered to be the most histologically relevant will be shown graphically and analysed in detail in this study. Details of all ten loadings can be found in the Supporting Information.

H&E stained sections were used to assist in the interpretation of the different tissue component loadings by correlating their relative positions within the Raman map. A linear combination of preselected reference biomolecules was fitted against each PC to identify the biomolecules that contribute most to the loading.

**Figure 2:**
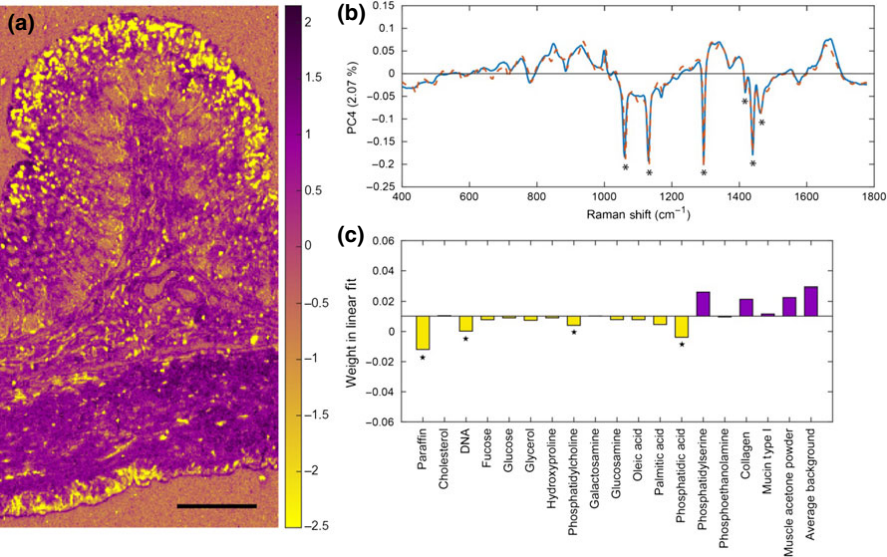
Principal component analysis results of paraffin contamination. (a) False‐coloured Raman map depicting the localization of paraffin contamination produced by assigning a user defined colour for the pixels corresponding to PC4. As indicated by the colour intensity scale bar (range −2.5 to +2.1) in this case intense yellow pixels correspond to strong paraffin signals. Intense purple pixels signify regions free of paraffin contamination but rich in other biological signals (length scale bar: 200 μm). (b) Principal component loading (blue) with reference linear model fit (orange). The strong negative peaks (troughs) in PC4, shown as yellow pixels in the map, have high magnitude and negative sign (asterisks) and match closely features of the reference paraffin spectrum included in the linear fit of all reference spectra (shown alongside, dotted line). (c) The weight of each chemical reference spectra used to generate the linear fit to PC4 showing the major contributing chemical species. Attention is drawn to the size of the bar in the chart rather than its positive or negative sign. Star symbols indicate paraffin and paraffin‐like signals from phospholipids showing that in the presence of paraffin the lipid data cannot be unambiguously recovered.

The most striking of all PCs was PC4 – characterized by a multitude of intense sharp peaks found in the negative region of the loading and assigned a yellow pixel colour. These features are akin to the unique features of paraffin wax used to embed the tissue. This demonstrates that despite attempts at rigorous paraffin removal in xylene (40 mins of total exposure with agitation), paraffin was well retained within the tissue. Paraffin was sparsely present across the whole tissue section but very strongly retained in the outermost mucosal edge (Figure 2a). Raman imaging facilitates easy identification of paraffin contamination via sharp intense paraffin peaks at 1062, 1132, 1294 and 1439 cm^−1^ in PC4 (Figure 2b). The linear reference fit indicates that PC4 is primarily composed of paraffin, but also exhibits the presence of some of the major phospholipids and some nucleic acids (Figure 2c). No other chemical contaminants were detected.

**Figure 3:**
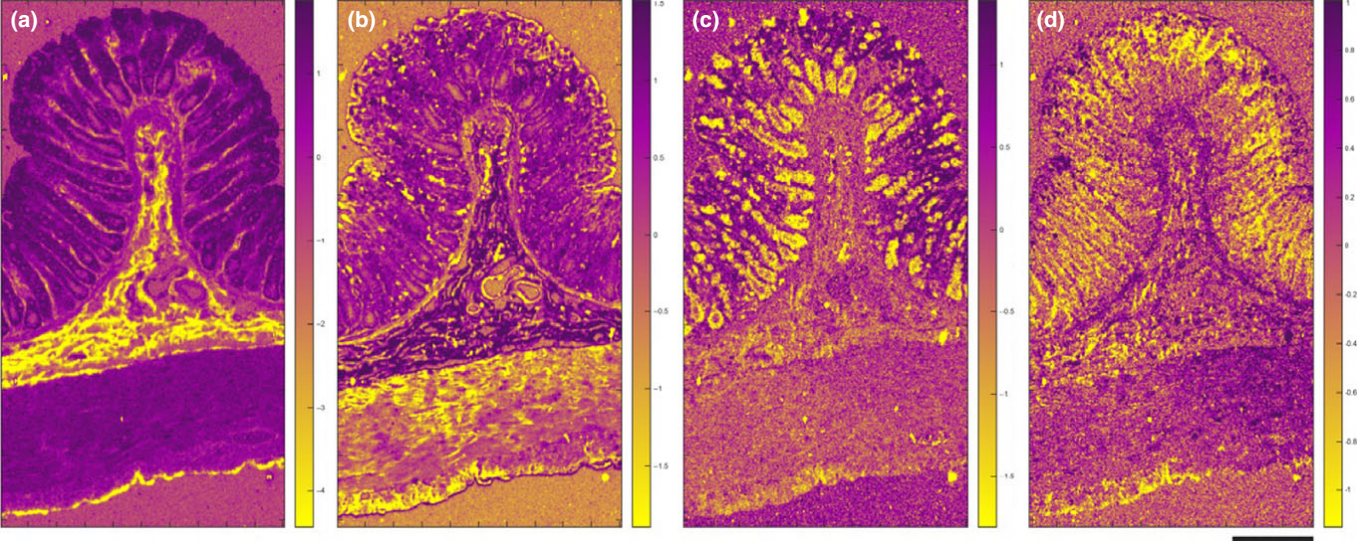
Raman false‐coloured images colour coded by the associated PC loadings and tentatively assigned to specific histological regions within the colon: (a) PC3: submucosa, lamina propria and serosa, (b) PC5: muscle, (c) PC6: mucin in goblet cells and (d) PC8: nuclei (scale: 200 μm). Note that the images are not heat maps and that the intensity of the purple or the yellow colours indicates the intensity of the component signals either positive or negative, respectively. Dark colours do not indicate the absence of signal but rather the presence of other strong signals of a different chemical type and spectral signature.

**Figure 4:**
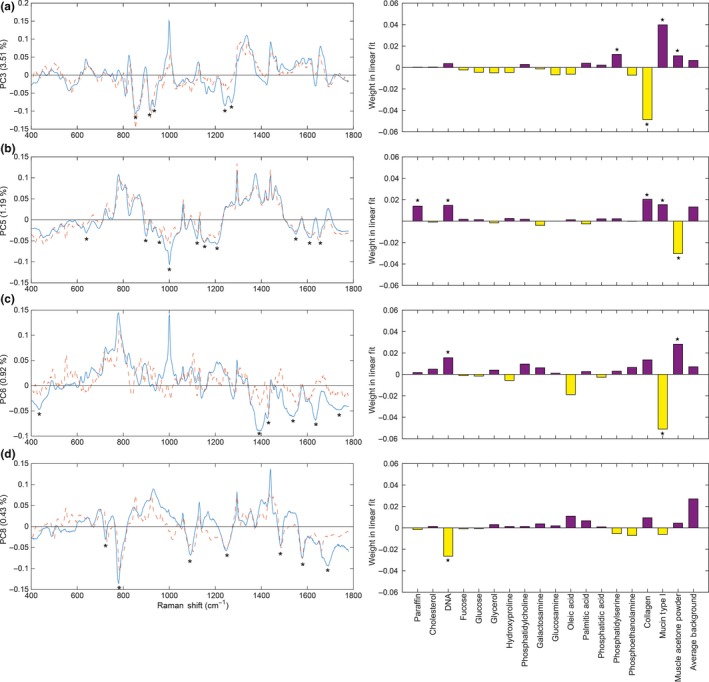
Loadings of principal components (blue): (a) PC3, (b) PC5, (c) PC6 and (d) PC8, each superimposed with a linear fit (orange) of the best matching molecular reference. Asterisks indicate the most significant molecular reference (right) alongside the associated spectral features (left). Note in the right‐hand panels that the purple and yellow colour coding of the positive or negative contribution of each reference species to the linear fit to the principle component has been preserved and is in accord with Figure 3.

The localization of PC3 coincides well with the lamina propria, submucosa and the serosa (Figure 3a). PC3 describes 3.51% of the total variance and portrays the main difference between collagen components (negative) and combined muscle/mucin/paraffin (positive) signals; this is described in the linear reference combination (Figure 4a). Intense peaks corresponding to proline and hydroxyproline were identified at 852, 916 and 933 cm^−1^. The positions of the spectrum amide bands are critical to determining the secondary structure of the proteins. The intense amide III peaks at 1242 cm^−1^ and 1268 cm^−1^ correspond to the triple helical structure of collagen (Gąsior‐Głogowska *et al*. 2010; Nguyen *et al*. 2012). The linear fit coincides well with the first two hydroxyproline peaks.

PC5 accounts for 1.19% of the total variance and depicts the main difference between muscle (negative) and combined paraffin/DNA/collagen/mucin (positive) signals (Figure 3b). The distribution of the loading covers the muscularis propria, muscularis mucosa as well as blood vessels in the submucosa. The most intense peak at 1002 cm^−1^ is attributed to the ring breathing mode of phenylalanine and is a ubiquitous proteinaceous constituent of biological tissue. Peaks at 638 and 1614 cm^−1^ (tyrosine), 1208 and 1550 cm^−1^ (phenylalanine, tryptophan) are all assigned to aromatic amino acids. Other protein‐associated peaks include 1121 and 1154 cm^−1^ (C‐C, C‐N stretch of proteins) and 1652 cm^−1^ (amide I). Some carbohydrate and lipid signals were also detected at 900 cm^−1^ and 955 cm^−1^ respectively (Figure 4b). Some of these peaks have previously been associated with actin and myosin components of muscle tissue (Barrett *et al*. 1978; Carew *et al*. 1983; de Jong *et al*. 2002). The linear reference molecule fit was best described by muscle acetone powder which fits well with the spatial localization of the loading within the map.

The localization of PC6 is in good agreement with goblet cell distribution within the H&E stained section (Figure 3c). PC6 is attributed to 0.92% of the variance and depicts the difference between mucin (negative) signals and combined DNA/muscle (positive) signals (Figure 4c). The most intense peak at 1390 cm^−1^ has been tentatively assigned to *N*‐acetyl‐galactosamine and *N*‐acetyl‐glucosamine as these are the most abundant sugar groups within mucins (Ashton *et al*. 2013). Other associated peaks were at 1426 cm^−1^ (deoxyribose), 1532 cm^−1^ (C=O) and 1635 cm^−1^ (amide I). Lipid peaks were detected at 435 and 1730 cm^−1^. The linear reference fit indicates that PC6 is positively characterized by nucleic acid and muscle signals, and negatively characterized by mucin signals. The overall fit to the loading, however, is poor in the high‐wavenumber region. A much better fit is obtained for the nucleic acid (721 and 778 cm^−1^) and muscle (1002 cm^−1^) associated signals; however, no mucin related peaks were observed in the fit (1300–1800 cm^−1^). The reliability of this fit to mucin is therefore questionable.

PC8 describes 0.43% of the total variation in the data and appears to capture the nucleic acid signals (Figure 3d). Nucleic acid signals are most abundant within the mucosa due to the presence of a very dense cell population in comparison to other layers of the colon (Figure 1b). One of the most characteristic peaks in this loading is at 778 cm^−1^, corresponding to uracil, thymine and cytosine. The remaining peaks all correspond to either DNA or RNA: 721 cm^−1^ (ring breathing modes of DNA), 1091 cm^−1^ (phosphodeoxy groups of nucleic acid backbone), 1246 cm^−1^ (asymmetric phosphate stretching modes from phosphodiester groups), 1484 cm^−1^ (nucleotide acid purine bases), 1577 cm^−1^ (guanine, adenine) and 1691 cm^−1^ (C=O group of bases). The linear reference fit indicates the loading is primarily composed of nucleic acids, which is in good agreement with the peak assignments and localization of the loading (Figure 4d).

**Figure 5:**
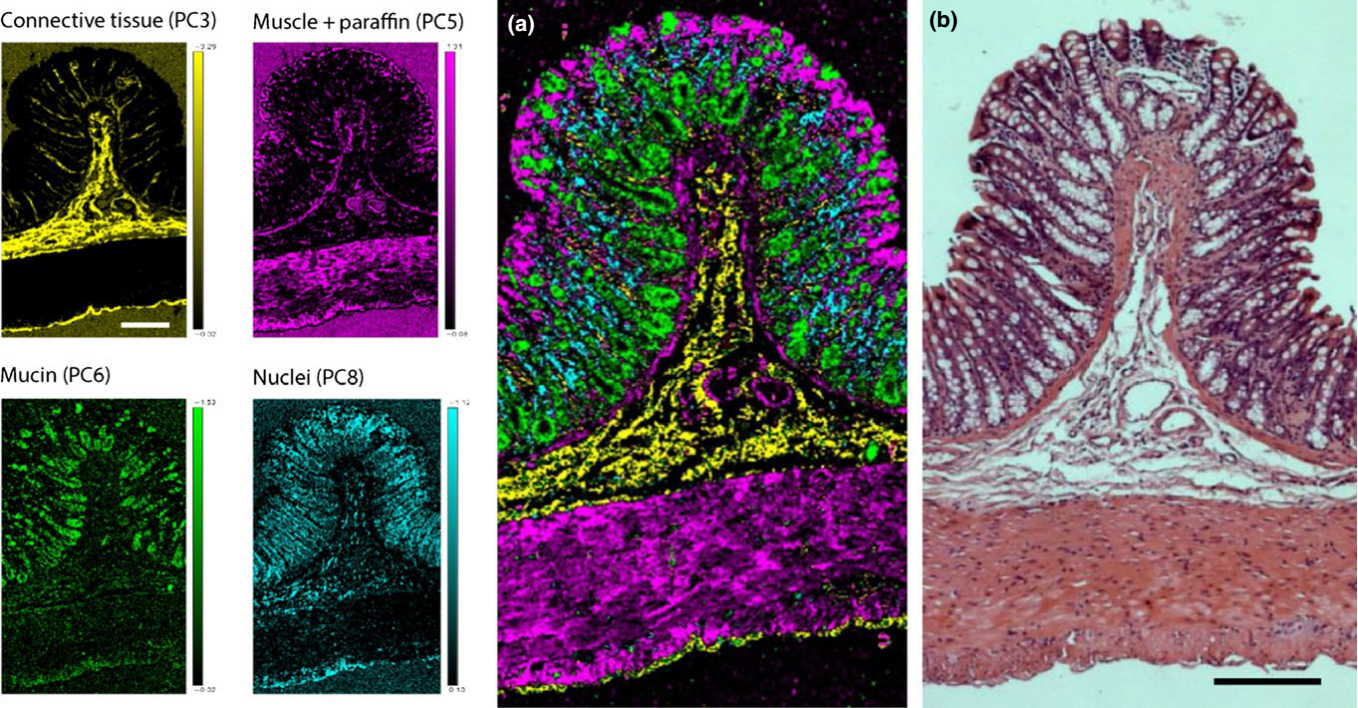
Comparison of H&E against a Raman false‐coloured composite score image. (a) PCA composite score image generated using WiRE 4 software encompassing the individual PC scores characteristic of the unique biochemical signatures of the different anatomical layers of the colon (scale: 200 μm). With this method the dominating spectral and chemical properties of each individual pixel in the tissue map are united in a high‐definition pseudo‐stained image. (b) Full‐thickness H&E stained rat colon section (adjacent to the Raman‐imaged unstained section).

The characteristic loadings can be combined to produce a false‐coloured composite score image that results in superior contrast to the traditionally used H&E staining techniques (Figure 5).

### Spectral similarity maps

Having established the main anatomical regions that can be resolved using PCA we attempted to match some of the reference biomolecules expected to be abundant within these regions.

**Figure 6:**
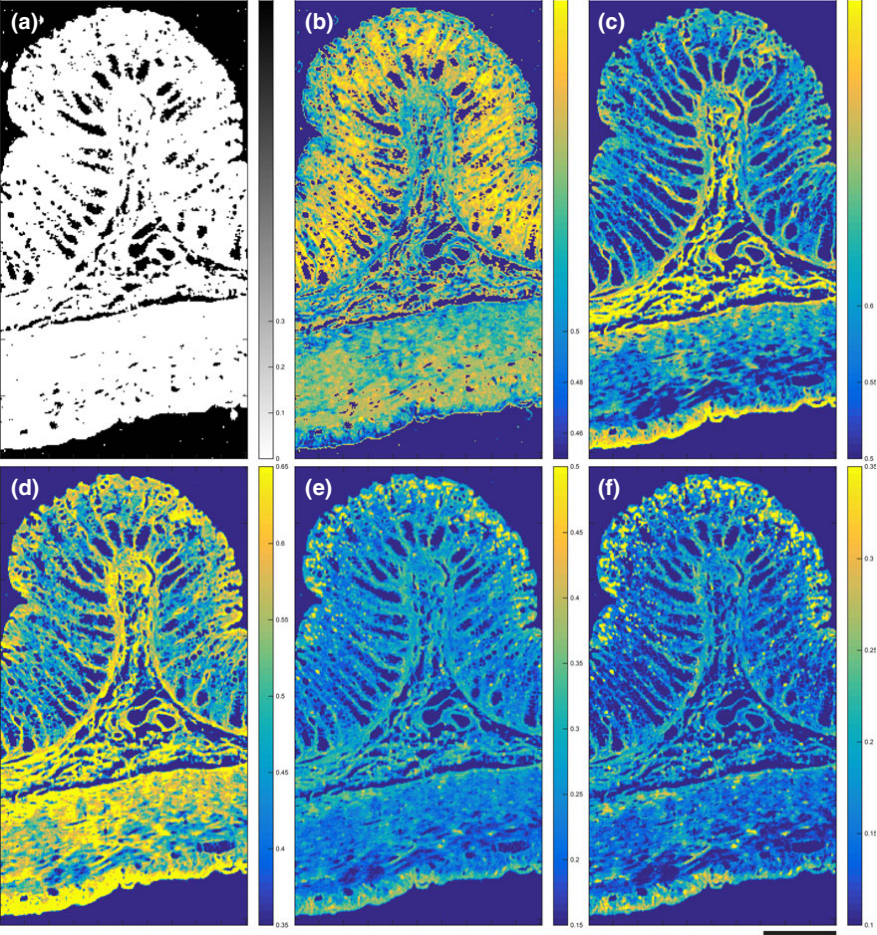
Spectral similarity maps of molecular references expected to be present within colon tissue. (a) Background mask, (b) DNA, (c) Collagen, (d) Muscle acetone powder, (e) Phosphatidylcholine, (f) Paraffin wax (scale: 200 μm). Note that in these images the intensity corresponds only to positive scores (within the range 0–1) with the brighter yellow colour indicating greater intensity and better spectral similarity to the reference sample.

Large areas of the Raman map do not contain biological tissue – most prominently the main lumen of the gastrointestinal tract. These areas still produce Raman signal, and can be spuriously labelled as being similar to reference spectra. As such, it is vital to determine and then discard those pixels that do not contain tissue before analysing the spectral similarity maps. This has been achieved by applying a background mask (Figure 6a).

Matching the similarity of a molecular reference to each pixel within the tissue map allows a direct visualization of the distribution and abundance based on pixel intensity. Use of similarity maps removes the need to tentatively assign peaks from Raman spectral databases. Computing the average of the best matching 100 tissue spectra can be used to reconstruct a best match spectrum. This enables easy identification of the most characteristic features associated with the biomolecular reference.

**Figure 7:**
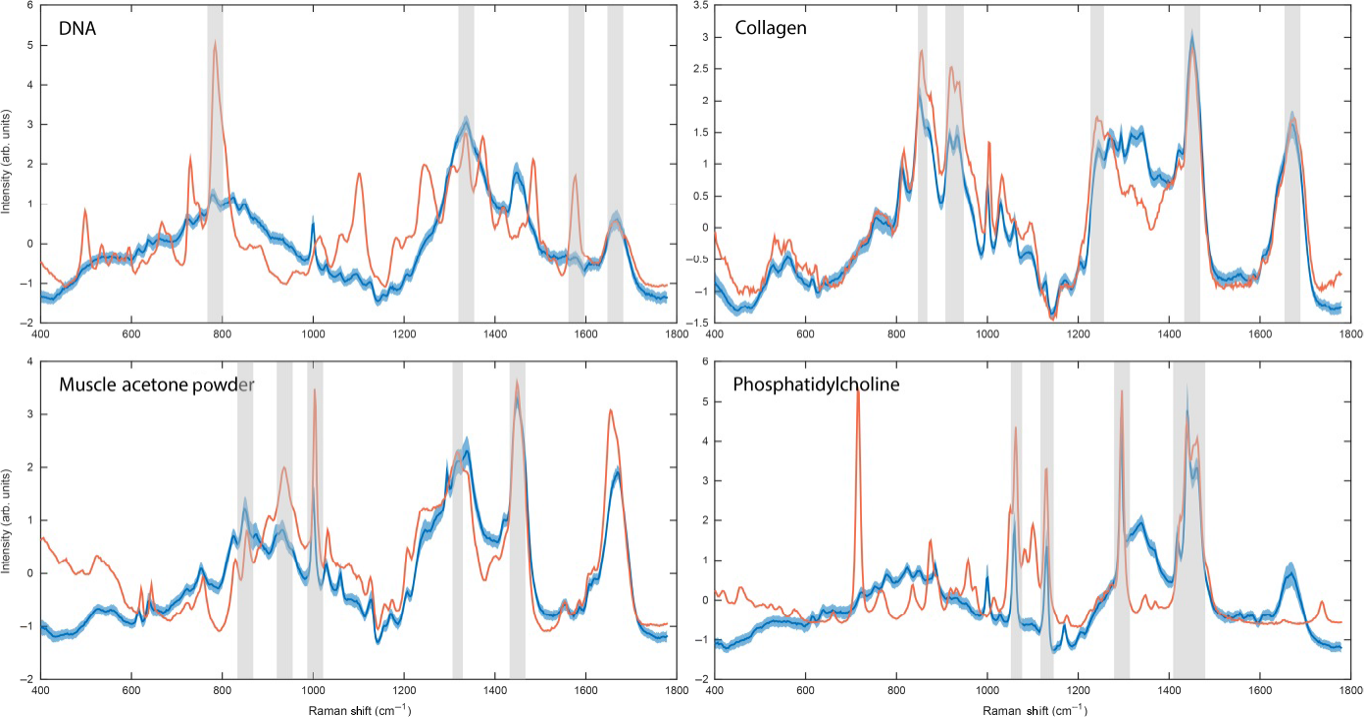
Reference spectra (orange) displayed alongside the average spectrum (blue) of the 100 best matching tissue map spectra along with their SD. Similarity scores of DNA θ = 0.65, collagen θ = 0.92, muscle acetone powder θ = 0.81 and phosphatidylcholine θ = 0.60 were achieved.

DNA followed an expected distribution across the tissue and was localized within the mucosa where the nuclear density is greatest. Similarity was also found across the submucosa where sparse lymphocytes are present as well as the nuclei of muscle cells within the muscularis propria (Figure 6b). The most characteristic peaks that best match the DNA reference spectrum are at 782, 1100, 1335, 1573 and 1662 cm^−1^ (Figure 7).

As expected, strong collagen signals were found to be abundant within the lamina propria and the submucosa. Of all the references, the collagen spectrum most closely matched tissue‐derived spectra with very high similarity between the reference and the average spectrum of the 100 best matching tissue spectra (θ = 0.92). Collagen associated spectra were also detected within the serosa and across the muscular regions – in particular at the interface between the circular muscle and the submucosa (Figure 6c). Proline and hydroxyproline amino acids unique to collagen were detected at 815, 854, 872, 920 and 935 cm^−1^ in the best matching tissue spectra. Other protein related peaks were detected at 533 and 561 cm^−1^, along with the amide III band at 1244 cm^−1^, CH deformation at 1450 cm^−1^ and 1674 cm^−1^ characteristic of amide I (Figure 7).

The muscle reference was found to localize within all muscular regions, but was also found within the submucosa and outermost luminal edge of the mucosa (Figure 6d). Characteristic peaks that were present within the average tissue spectrum and reference were particularly assigned to aromatic amino acids phenylalanine (620, 1002, 1030 cm^−1^), tyrosine (642, 828, 852 cm^−1^) and tryptophan (757, 1551 cm^−1^). Other protein related bands were at 935 cm^−1^ (proline, valine, collagen), 1124 cm^−1^ (C‐N protein vibration), 1315 cm^−1^ (CH_3_CH_2_ twisting mode) and 1447 cm^−1^ (CH_2_ bending mode of proteins) (Figure 7). A spectral similarity of θ = 0.81 was achieved.

The most abundant tissue phospholipid – phosphatidylcholine – co‐localized with pixel regions that were heavily contaminated with paraffin wax (Figure 6e,f). This is further corroborated by matching the reference spectrum with the average of the hundred most similar tissue spectra. Overlapping peaks were found at 1062, 1132, 1294, 1439 and 1460 cm^−1^ (Figure 7). The remaining references failed to match – this was detected by poor co‐localization within the map and/or an average tissue spectrum corresponding to the background.

## Discussion

Up until now there have been no previous attempts to provide a detailed biochemical characterization of the layers of healthy colon tissue following FFPE treatment. Only one other study has thus far been conducted on frozen human colon that was passively dried prior to Raman analysis. However, the selected spatial resolution was significantly lower than that in our study at a step size of 10 μm at the mucosal surface, compared to our step size of 2.8 μm (Krafft *et al*. 2008). Our results clearly indicate that Raman microspectroscopy can discern the biochemical difference from within each layer following extensive chemical processing – resulting in superior contrast to the currently universally used H&E stain (Figure 5).

Before conducting any biological analysis efforts were made to first identify any chemical contaminants which may interfere with the analysis. PCA revealed widespread paraffin contamination with a good linear fit to a paraffin reference. The distinct difference between true biological material and the added organic paraffin enabled easy identification of such contamination. The incompleteness of paraffin removal has been reported previously in several other studies (Faoláin *et al*. 2005; Nallala *et al*. 2015), and thus far, no mechanism for the complete removal of paraffin with any known deparaffinizing agent has been discovered. Discovery of a method for the complete removal of paraffin with the maintenance of biochemical signals and tissue morphology could have significant benefits in clinical diagnosis or in research. For example, prolonged incubation of tissue sections (~18 h) in hexane results in near‐complete removal of paraffin and contributes to a 30% increase in IHC staining (Faoláin *et al*. 2005). Significant wax retention is expected to compromise IHC results by blocking the presentation of antigens to antibodies. Our work further contributes to the current body of evidence that Raman analysis can provide the means to assess paraffin retention, which can potentially be used as a measure for wax removal efficacy and provide a means of quality control for optimized IHC staining. Hexane deparaffinization was not attempted here as it is likely to result in the leaching out of the remaining lipid signals.

Furthermore, clear identification of phospholipid components (such as phosphatidylcholine in this study) is hindered by paraffin retention – in the presence of paraffin, the signals from phospholipids cannot be unambiguously monitored. The obvious reason why the majority of all the lipid references tested were found to overlap with pixels containing paraffin is because of the overall structural similarity of fatty acyl groups and paraffin. To retrieve biochemical information on tissue phospholipid status, it will be essential to ensure close to complete paraffin removal from tissue sections prior to Raman mapping and reference matching. Given the strong diagnostic correlation between aberrant phospholipid metabolism and disease, and the prospects for therapy (e.g. cancer (Ackerstaff *et al*. 2003)) the value of Raman in monitoring and validating complete paraffin removal and recovering data on the remaining tissue phospholipids is self‐evident.

Collagen signals were easily identified using both unsupervised PCA as well as spectral similarity maps with the highest similarity score of θ = 0.92. Of the biologically relevant PCs the loading associated with collagen accounted for the higher proportion of the variance within the data set (3.51%) compared to other components. All collagens consist of a set of three polypeptide chains held together in a helical conformation via hydrogen bonding. Each polypeptide chain is characterized by a repeating Gly‐X‐Y sequence where X corresponds frequently to proline and Y to hydroxyproline (Shoulders & Raines 2009; Nguyen *et al*. 2012). The presence of hydroxyproline (rare in most other proteins) therefore makes collagen quite a unique molecular entity that is quite easily recognized. This might prove to be useful in the detection of collagen elevation associated with fibrostenotic IBD, for example. Using PCA, we were able to confine the collagen signals to the submucosa and serosa where connective tissue binds mesothelial cells to the outer longitudinal muscle. On the other hand, similarity maps exhibited some collagen‐like signals at the interface between the submucosa and the muscularis propria as well as the outermost edge of the longitudinal muscle. This is likely caused by the overlap of certain vibrational signals that are also present in muscle tissue. More importantly, the frequency shifts observed in the amide III bands are in agreement with shifts observed in unfixed/frozen tissue (Krafft *et al*. 2008), this suggests that formalin fixation did not impose any detrimental effects on the collagen components on this occasion.

Along with collagen, nucleic acid signals were also one of the easiest molecular entities to identify using both similarity maps and PCA. This demonstrates that nucleic acid signals can potentially be quantified with relative ease, facilitating studies in cellular differentiation and carcinogenesis. In this particular case, 24 hr formalin fixation and extensive downstream processing along with attempts at rigorous deparaffinization of a small 5‐mm‐by‐5‐mm rat colon resection had no adverse effects on the detection of nucleic acid signals.

Muscular regions were associated with abundant protein signals, in particular from the aromatic amino acids phenylalanine, tryptophan and tyrosine. This has previously been observed from frozen then passively dried colon tissue sections (Krafft *et al*. 2008). PCA shows that some muscle‐like signals were also present across the luminal edge of the mucosa (Figure 4b); however, the reference linear fit suggests that these signals are of lipid origin and are likely detected from the cytoplasmic components of the luminal epithelial cells (Figure 5b). This is further corroborated by the H&E stained image as the luminal epithelial cells have a clearly visible cytoplasmic component (Figure 1b).

The identification of mucin in FFPE tissue suggests that mucin was not completely removed during chemical processing. However, its retention within goblet cells is also likely to be affected by the extent of rough handling and overall disturbance of the mucosa. Intestinal mucins are composed of approximately 80% carbohydrate side chains bound to a protein core. Five different carbohydrate moieties: *N*‐acetyl‐galactosamine, *N*‐acetyl‐glucosamine, fucose, galactose and sialic acid are arranged in side chains and trace amounts of mannose and sulphates have also been found (Fogg *et al*. 1996; Johansson *et al*. 2011). Using PCA, we were able to identify the large carbohydrate content of mucins and therefore isolate the goblet cells (Figure 5c). Previous Raman studies of mucin molecules have revealed intense signals originating primarily from the carbohydrate moieties and not so much from the proteins (Ashton *et al*. 2013). The lipid signals detected are a likely result of the presence of polar/neutral lipids and glycolipids that confer surface hydrophobicity, as well as viscous and lubricant properties to mucus (Lichtenberger 1995). Detection of mucins during Raman analysis therefore provides an additional scope for assessing tissue homoeostasis and early metastatic potential (Bresalier *et al*. 1996; Kufe 2009; Mekenkamp *et al*. 2012).

Conversely some of the tissue components were not easily distinguished such as the epithelial cells lining the inner crypts and the lymphocytes within the lamina propria. The predominant signals from both of these cell types were of nuclear origin as the extremely compact nature of the rat mucosa leaves very little visible cell cytoplasm (Figure 1b). By opting for a near infrared 785 nm wavelength and a line geometry, we were able to not only reduce the overall tissue autofluorescence but also reduce the likelihood of photothermally degrading the sample because of the dispersion of the laser density across a line. The spatial resolution of a Raman image is dependent on a number of different parameters: (i) objective, (ii) laser wavelength, (iii) laser geometry and (iv) step size used to acquire the image (Pascut *et al*. 2011). Using a 785 nm laser line for mapping at a 2.8 μm step size would enable features larger than 2.8 μm to be resolved; features below this such as the compact cytoplasm will need to be resolved by sampling at a smaller step size. To distinguish the different cell types, we would need to capture the protein expression profiles of each cell, and hence, the cytoplasm would be a crucial feature. It is likely that the combined signal from both the nucleus and cytoplasm may be crucial for the discrimination of different cell types. We were therefore unable to distinguish the finer features of the colon such as the stem cells at the base of the crypts. Raman imaging using higher spatial resolution may be required to reveal such fine anatomical structures. This may be realized by employing an objective with a higher NA, shorter laser wavelength, for example 532 nm, laser spot configuration and a smaller step size.

Our ability to successfully detect specific biomolecular references strongly depends on the abundance and purity of the molecular reference. In this case, a single band does not correspond to a single chemical entity but instead to the vibrational state of a given chemical bond, which may be shared across several different molecules. It is therefore important to consider that although levels of the reference spectra may increase in some specific tissue regions that were matched, there will still be many other contributions from other biochemical entities; hence, spectral similarity and linear fit of PCA may not perform well where there is wide chemical diversity. We have, however, observed superior performance of more complex reference standards such as collagen, mucin and muscle over samples of their individual molecular subcomponents, for example amino acids and sugars. This was observed on numerous occasions where collagen outperformed hydroxyproline (Figure 4a) and mucin outperformed the most abundant mucin sugars *N*‐acetyl glucosamine and *N*‐acetyl galactosamine (Figure 4c). Furthermore, applying PCA led to superior results over similarity maps without the need for selecting biomolecular references.

This study demonstrates that Raman microspectroscopy can be successfully used to discern all the major anatomical layers of colon tissue processed with conventional FFPE techniques. Raman is therefore a suitable technique for tissues that have undergone processing using the standard pathological workflow. There is an overall good agreement in the Raman spectral assignments of FFPE colon tissue layers and chemically untreated biomolecular references. This confirms that chemical processing does not lead to an unacceptable distortion in the spectral profiles of tissues. Additionally, mucin signals were shown to be well retained following extensive chemical processing emphasizing the desirability of gentler handling of the fresh tissue specimens to prevent mucin leakage and preserve any signals that may be of diagnostic use. Furthermore, we have shown that the detection of specific biomolecular references within the tissue requires careful selection of references. The spectral profiles established for each layer can now be used in subsequent rat studies where there are pathological deviations from the normal condition.

## Conclusion

Within this study, we have described the application of Raman microspectroscopy to characterize all the major layers of colon tissue in samples that have undergone standard pathology laboratory processing. Despite widespread paraffin contamination, good resolution was achieved and this enabled easy identification of all major layers of the colon along with a complete characterization of the associated biochemical signals. The morphological and biochemical information obtained from healthy colons can provide comparisons to colons in different disease models. Extensive chemical preprocessing in formalin, alcohol and xylene did not impede the quality of the spectral signatures in our study; however, paraffin contamination was detected and needs to be resolved to improve the results further. Through the use of PCA, composite score images provided a superior contrast to the conventional H&E‐stained sections. The anatomical layer identification was confirmed by similarity maps correlating to the abundance of known chemical components of each layer. Our results show that Raman analysis can be used as a complementary technique facilitating the analysis of tissue on both a morphological and metabolic level in animal models and humans.

## Conflict of interest

There are no known conflict of interests.

## Funding source

This study was funded through the UCL Impact PhD Scheme  (to RG) with support from Renishaw Spectroscopy Division (Renishaw plc), the Medical Research Council (to RG, reference MC_PC_14118 v.2 PO 050471026) and the Engineering and Physical Sciences Research Council (to ATM, reference EP/M506448/1). Dr Manuel Rodriguez‐Justo is supported by the UCLH/UCL NIHR Biomedical Research Centre. We would like to thank UCLH Advanced Diagnostics and UCLH Histopathology for providing help with tissue processing, in particular Mr Keith Miller for arranging training in histopathology. We would also like to extend our gratitude to Mr Tim Smith, Dr Tim Batten and Dr Yan Wong for instrumental support.

## Supporting information


**Figure S1.** PC loadings 1–10. The first 10 loadings explain 82% of the total variance in the dataset.

## References

[iep12194-bib-0001] Ackerstaff E. , Glunde K. & Bhujwalla Z.M. (2003) Choline phospholipid metabolism: a target in cancer cells? J. Cell. Biochem. 90, 525–533. 14523987 10.1002/jcb.10659

[iep12194-bib-0002] Ashton L. , Pudney P.D.A. , Blanch E.W. & Yakubov G.E. (2013) Understanding glycoprotein behaviours using Raman and Raman optical activity spectroscopies: characterising the entanglement induced conformational changes in oligosaccharide chains of mucin. Adv. Colloid Interface Sci. 199–200, 66–77. 10.1016/j.cis.2013.06.005 23859222

[iep12194-bib-0003] Barrett T.W. , Peticolas W.L. & Robson R.M. (1978) Laser Raman light‐scattering observations of conformational changes in myosin induced by inorganic salts. Biophys. J. 23, 349–358. 698341 10.1016/S0006-3495(78)85454-X PMC1473530

[iep12194-bib-0004] Beljebbar A. , Bouché O. , Diébold M.D. *et al* (2009) Identification of Raman spectroscopic markers for the characterization of normal and adenocarcinomatous colonic tissues. Crit. Rev. Oncol. Hematol. 72, 255–264. 19819161 10.1016/j.critrevonc.2009.09.004

[iep12194-bib-0005] Bi X. , Walsh A. , Mahadevan‐Jansen A. & Herline A. (2011) Development of spectral markers for the discrimination of ulcerative colitis and Crohn's disease using Raman spectroscopy. Dis. Colon Rectum 54, 48–53. 21160313 10.1007/DCR.0b013e3181fcf68d

[iep12194-bib-0006] Bielecki C. , Bocklitz T.W. , Schmitt M. *et al* (2012) Classification of inflammatory bowel diseases by means of Raman spectroscopic imaging of epithelium cells. J. Biomed. Opt. 17, 076030. 22894513 10.1117/1.JBO.17.7.076030

[iep12194-bib-0007] Bonnier F. & Byrne H.J. (2012) Understanding the molecular information contained in principal component analysis of vibrational spectra of biological systems. Analyst 137, 322. 22114757 10.1039/c1an15821j

[iep12194-bib-0008] Bresalier R. , Ho S. , Schoeppner H. *et al* (1996) Enhanced sialylation of mucin‐associated carbohydrate structures in human colon cancer metastasis. Gastroenterology 110, 1354–1367. 8613039 10.1053/gast.1996.v110.pm8613039

[iep12194-bib-0009] Butler H.J. , Ashton L. , Bird B. *et al* (2016) Using Raman spectroscopy to characterise biological materials. Nat. Protoc. 11, 1–47. 26963630 10.1038/nprot.2016.036

[iep12194-bib-0010] Carew E.B. , Stanley H.E. , Seidel J.C. & Gergely J. (1983) Studies of myosin and its proteolytic fragments by laser Raman spectroscopy. Biophys. J. 44, 219–224. 6360227 10.1016/S0006-3495(83)84294-5 PMC1434820

[iep12194-bib-0011] Cassidy M.M. , Lightfoot F.G. , Grau L.E. *et al* (1980) Effect of bile salt‐binding resins on the morphology of rat jejunum and colon. Dig. Dis. Sci. 25, 504–512. 7389538 10.1007/BF01315212

[iep12194-bib-0012] Chowdary M.V.P. , Kumar K.K. , Thakur K. *et al* (2007) Discrimination of normal and malignant mucosal tissues of the colon by Raman spectroscopy. Photomed. Laser Surg. 25, 269–274. 17803383 10.1089/pho.2006.2066

[iep12194-bib-0013] Devpura S. , Thakur J.S. , Poulik J.M. , Rabah R. , Naik V.M. & Naik R. (2013) Raman spectroscopic investigation of frozen and deparaffinized tissue sections of pediatric tumors: neuroblastoma and ganglioneuroma. J. Raman Spectrosc. 44, 370–376.

[iep12194-bib-0014] Dieleman L.A. , Hoentjen F. , Qian B.‐F. *et al* (2004) Reduced ratio of protective versus proinflammatory cytokine responses to commensal bacteria in HLA‐B27 transgenic rats. Clin. Exp. Immunol. 136, 30–39. 15030511 10.1111/j.1365-2249.2004.02410.x PMC1808999

[iep12194-bib-0015] Donaldson D.S. , Bradford B.M. , Artis D. & Mabbott N.A. (2015) Reciprocal regulation of lymphoid tissue development in the large intestine by IL‐25 and IL‐23. Mucosal Immunol. 8, 582–595. 25249168 10.1038/mi.2014.90 PMC4424384

[iep12194-bib-0016] Fan H. , Qiu M.‐Y. , Mei J.‐J. , Shen G.‐X. , Liu S.‐L. & Chen R. (2005) Effects of four regulating‐intestine prescriptions on pathology and ultrastructure of colon tissue in rats with ulcerative colitis. World J. Gastroenterol. 11, 4800–4806. 16097047 10.3748/wjg.v11.i31.4800 PMC4398725

[iep12194-bib-0017] Faoláin E.O. , Hunter M.B. , Byrne J.M. *et al* (2005) Raman spectroscopic evaluation of efficacy of current paraffin wax section dewaxing agents. J. Histochem. Cytochem. 53, 121–129. 15637345 10.1177/002215540505300114

[iep12194-bib-0018] Fogg F.J. , Hutton D.A. , Jumel K. , Pearson J.P. , Harding S.E. & Allen A. (1996) Characterization of pig colonic mucins. Biochem. J. 316(Pt 3), 937–942. 8670173 10.1042/bj3160937 PMC1217439

[iep12194-bib-0019] Ganguly K. , Kulkarni A.R. & Aminabhavi T.M. (2015) In vitro cytotoxicity and in vivo efficacy of 5‐fluorouracil‐loaded enteric‐coated PEG‐crosslinked chitosan microspheres in colorectal cancer therapy in rats. Drug Deliv. 1–14. doi: 10.3109/10717544.2015.1089955. 10.3109/10717544.2015.1089955 PMC12519578 26394122

[iep12194-bib-0020] Gąsior‐Głogowska M. , Komorowska M. , Hanuza J. , Ptak M. & Kobielarz M. (2010) Structural alteration of collagen fibres–spectroscopic and mechanical studies. Acta Bioeng. Biomech. 12, 55–62. 21361257

[iep12194-bib-0021] Humphries A. & Wright N.A. (2008) Colonic crypt organization and tumorigenesis. Nat. Rev. Cancer 8, 415–424. 18480839 10.1038/nrc2392

[iep12194-bib-0022] Johansson M.E.V. , Ambort D. , Pelaseyed T. *et al* (2011) Composition and functional role of the mucus layers in the intestine. Cell. Mol. Life Sci. 68, 3635–3641. 21947475 10.1007/s00018-011-0822-3 PMC11114784

[iep12194-bib-0023] de Jong B.W.D. , Bakker Schut T.C. , Wolffenbuttel K.P. , Nijman J.M. , Kok D.J. & Puppels G.J. (2002) Identification of bladder wall layers by Raman spectroscopy. J. Urol. 168, 1771–1778. 12352357 10.1097/01.ju.0000030059.28948.c6

[iep12194-bib-0024] Klein K. , Gigler A.M. , Aschenbrenner T. *et al* (2012) Label‐free live‐cell imaging with confocal Raman microscopy. Biophys. J. 102, 360–368. 22339873 10.1016/j.bpj.2011.12.027 PMC3260693

[iep12194-bib-0025] Krafft C. , Codrich D. , Pelizzo G. & Sergo V. (2008) Raman and FTIR microscopic imaging of colon tissue: a comparative study. J. Biophotonics 1, 154–169. 19343646 10.1002/jbio.200710005

[iep12194-bib-0026] Kufe D.W. (2009) Mucins in cancer: function, prognosis and therapy. Nat. Rev. Cancer 9, 874–885. 19935676 10.1038/nrc2761 PMC2951677

[iep12194-bib-0027] Li S. , Chen G. , Zhang Y. *et al* (2014) Identification and characterization of colorectal cancer using Raman spectroscopy and feature selection techniques. Opt. Express 22, 25895–25908. 25401621 10.1364/OE.22.025895

[iep12194-bib-0028] Lichtenberger L.M. (1995) The hydrophobic barrier properties of gastrointestinal mucus. Annu. Rev. Physiol. 57, 565–583. 7778878 10.1146/annurev.ph.57.030195.003025

[iep12194-bib-0029] Lloyd G.R. , Wood J. , Kendall C. , Cook T. , Shepherd N. & Stone N. (2012) Histological imaging of a human colon polyp sample using Raman spectroscopy and self organising maps. Vib. Spectrosc. 60, 43–49.

[iep12194-bib-0030] Lui H. , Zhao J. , McLean D. & Zeng H. (2012) Real‐time Raman spectroscopy for in vivo skin cancer diagnosis. Cancer Res. 72, 2491–2500. 22434431 10.1158/0008-5472.CAN-11-4061

[iep12194-bib-0031] Maier J.S. & Treado P.J. (2004) Raman molecular chemical imaging: 3D Raman using deconvolution In: Optics East, pp. 98–105 (ed. Cullum B.M.), Philadelphia, PA: International Society for Optics and Photonics.

[iep12194-bib-0032] Mavarani L. , Petersen D. , El‐Mashtoly S.F. *et al* (2013) Spectral histopathology of colon cancer tissue sections by Raman imaging with 532 nm excitation provides label free annotation of lymphocytes, erythrocytes and proliferating nuclei of cancer cells. Analyst 138, 4035–4039. 23733134 10.1039/c3an00370a

[iep12194-bib-0033] Mekenkamp L.J.M. , Heesterbeek K.J. , Koopman M. *et al* (2012) Mucinous adenocarcinomas: poor prognosis in metastatic colorectal cancer. Eur. J. Cancer 48, 501–509. 22226571 10.1016/j.ejca.2011.12.004

[iep12194-bib-0034] Molckovsky A. , Song L.‐M.W.K. , Shim M.G. , Marcon N.E. & Wilson B.C. (2003) Diagnostic potential of near‐infrared Raman spectroscopy in the colon: differentiating adenomatous from hyperplastic polyps. Gastrointest. Endosc. 57, 396–402. 12612529 10.1067/mge.2003.105

[iep12194-bib-0035] Nallala J. , Diebold M.‐D. , Gobinet C. *et al* (2014) Infrared spectral histopathology for cancer diagnosis: a novel approach for automated pattern recognition of colon adenocarcinoma. Analyst 139, 4005–4015. 24932462 10.1039/c3an01022h

[iep12194-bib-0036] Nallala J. , Lloyd G.R. & Stone N. (2015) Evaluation of different tissue de‐paraffinization procedures for infrared spectral imaging. Analyst 140, 2369–2375. doi: 10.1039/C4AN02122C. 25671463 10.1039/c4an02122c

[iep12194-bib-0037] Nguyen T.T. , Gobinet C. , Feru J. , Pasco S.B. , Manfait M. & Piot O. (2012) Characterization of type I and IV collagens by Raman microspectroscopy: identification of spectral markers of the dermo‐epidermal junction. Spectrosc. Ann. Int. J. 27, 421–427.

[iep12194-bib-0038] Nightingale J.M. (2001) Management of patients with a short bowel. World J. Gastroenterol. 7, 741–751. 11819867 10.3748/wjg.v7.i6.741 PMC4695587

[iep12194-bib-0039] Pascut F.C. , Goh H.T. , Welch N. , Buttery L.D. , Denning C. & Notingher I. (2011) Noninvasive detection and imaging of molecular markers in live cardiomyocytes derived from human embryonic stem cells. Biophys. J. 100, 251–259. 21190678 10.1016/j.bpj.2010.11.043 PMC3010010

[iep12194-bib-0040] Piva J.A.A.C. , Silva J.L.R. , Raniero L. , Martin A.A. , Bohr H.G. & Jalkanen K.J. (2011) Overview of the use of theory to understand infrared and Raman spectra and images of biomolecules: colorectal cancer as an example. Theor. Chem. Acc. 130, 1261–1273.

[iep12194-bib-0041] Rijnierse A. , Koster A.S. , Nijkamp F.P. & Kraneveld A.D. (2006) Critical role for mast cells in the pathogenesis of 2,4‐dinitrobenzene‐induced murine colonic hypersensitivity reaction. J. Immunol. 176, 4375–4384. 16547276 10.4049/jimmunol.176.7.4375

[iep12194-bib-0042] Shimma S. , Sugiura Y. , Hayasaka T. , Hoshikawa Y. , Noda T. & Setou M. (2007) MALDI‐based imaging mass spectrometry revealed abnormal distribution of phospholipids in colon cancer liver metastasis. J. Chromatogr. B Analyt. Technol. Biomed. Life Sci. 855, 98–103. 10.1016/j.jchromb.2007.02.037 17369111

[iep12194-bib-0043] Shoulders M.D. & Raines R.T. (2009) Collagen structure and stability. Annu. Rev. Biochem. 78, 929–958. 19344236 10.1146/annurev.biochem.77.032207.120833 PMC2846778

[iep12194-bib-0044] Srinivasan M. , Sedmak D. & Jewell S. (2002) Effect of fixatives and tissue processing on the content and integrity of nucleic acids. Am. J. Pathol. 161, 1961–1971. 12466110 10.1016/S0002-9440(10)64472-0 PMC1850907

[iep12194-bib-0045] Stĕpánková R. , Tlaskalová‐Hogenová H. , Sinkora J. , Jodl J. & Fric P. (1996) Changes in jejunal mucosa after long‐term feeding of germfree rats with gluten. Scand. J. Gastroenterol. 31, 551–557. 8789893 10.3109/00365529609009127

[iep12194-bib-0046] Taketani A. , Ishigaki M. , Andriana B.B. & Sato H. (2014) Raman endoscopy for real time monitoring of anticancer drug treatment in colorectal tumors of live model mice In: SPIE BiOS, p. 89510O (ed. Coté G.L.), San Francisco, CA: International Society for Optics and Photonics.

[iep12194-bib-0047] Widjaja E. , Zheng W. & Huang Z. (2008) Classification of colonic tissues using near‐infrared Raman spectroscopy and support vector machines. Int. J. Oncol. 32, 653–662. 18292943

[iep12194-bib-0048] Yaziji H. & Barry T. (2006) Diagnostic immunohistochemistry: what can go wrong? Adv. Anat. Pathol. 13, 238–246. 16998317 10.1097/01.pap.0000213041.39070.2f

