# Supplementary material for: Label‐free Raman spectroscopic imaging to extract morphological and chemical information from a formalin‐fixed, paraffin‐embedded rat colon tissue section
Source: Int J Exp Pathol. 2016 Sep 1;97(4):337–50. doi: 10.1111/iep.12194 (PMC5061758; doi:10.1111/iep.12194)
Supplement: Supplementary file 1 — Figure S1. PC loadings 1–10. The first 10 loadings explain 82% of the total variance in the dataset. [file IEP-97-337-s001.docx]

**7. Supplementary Information**


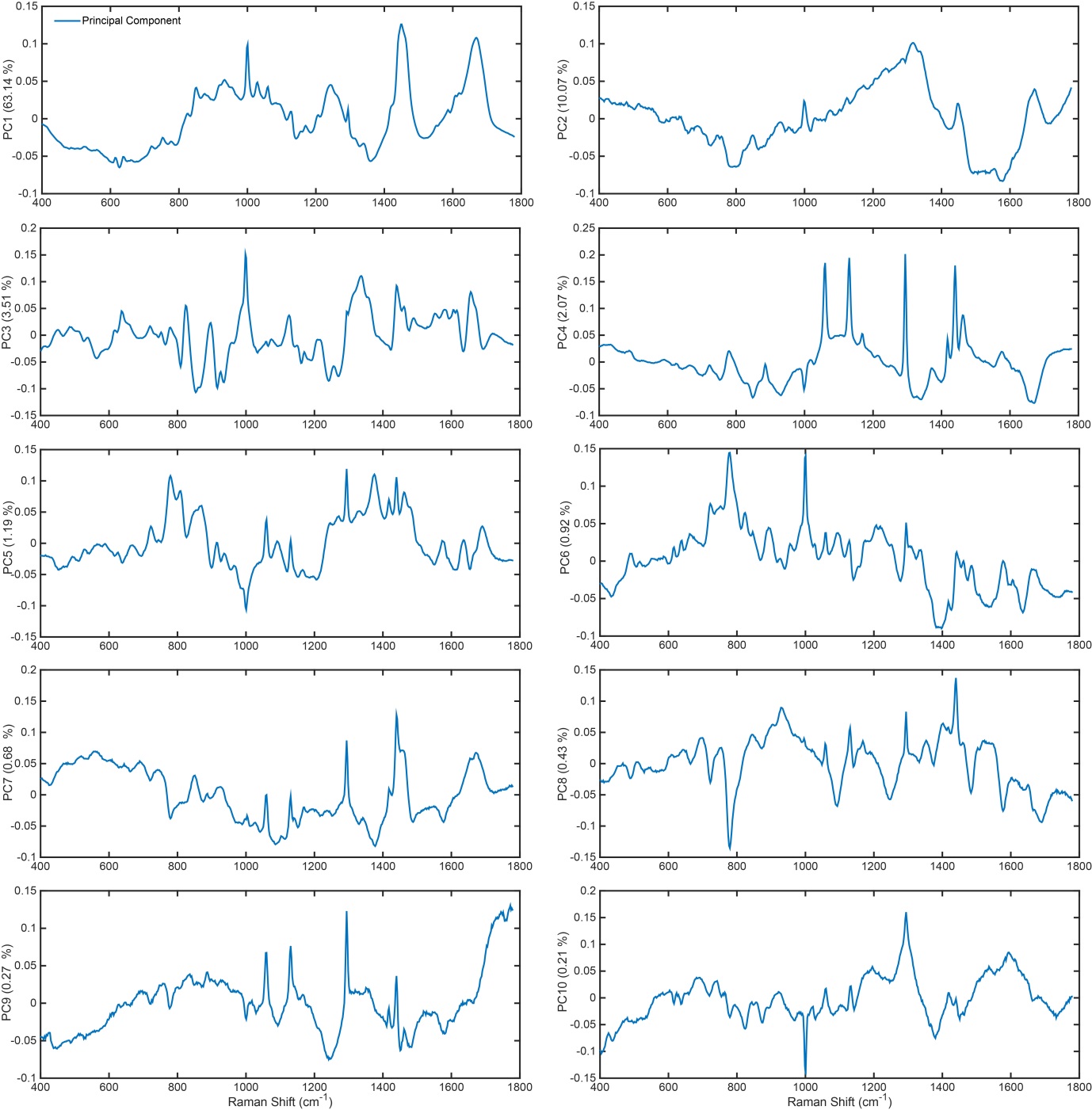


*Figure 1: PC loadings 1-10. The first 10 loadings explain 82 % of the total variance in the dataset.*
